# The Mitochondrial Genome of the Prasinophyte *Prasinoderma coloniale* Reveals Two *Trans*-Spliced Group I Introns in the Large Subunit rRNA Gene

**DOI:** 10.1371/journal.pone.0084325

**Published:** 2013-12-26

**Authors:** Jean-François Pombert, Christian Otis, Monique Turmel, Claude Lemieux

**Affiliations:** 1 Department of Biological and Chemical Sciences, Illinois Institute of Technology, Chicago, Illinois, United States of America; 2 Institut de Biologie Intégrative et des Systèmes, Département de Biochimie, de Microbiologie et de Bio-informatique, Université Laval, Québec, Québec, Canada; University of Toronto, Canada

## Abstract

Organelle genes are often interrupted by group I and or group II introns. Splicing of these mobile genetic occurs at the RNA level via serial transesterification steps catalyzed by the introns'own tertiary structures and, sometimes, with the help of external factors. These catalytic ribozymes can be found in *cis* or *trans* configuration, and although *trans*-arrayed group II introns have been known for decades, *trans*-spliced group I introns have been reported only recently. In the course of sequencing the complete mitochondrial genome of the prasinophyte picoplanktonic green alga *Prasinoderma coloniale* CCMP 1220 (Prasinococcales, clade VI), we uncovered two additional cases of *trans*-spliced group I introns. Here, we describe these introns and compare the 54,546 bp-long mitochondrial genome of *Prasinoderma* with those of four other prasinophytes (clades II, III and V). This comparison underscores the highly variable mitochondrial genome architecture in these ancient chlorophyte lineages. Both *Prasinoderma trans*-spliced introns reside within the large subunit rRNA gene (*rnl*) at positions where *cis*-spliced relatives, often containing homing endonuclease genes, have been found in other organelles. In contrast, all previously reported *trans*-spliced group I introns occur in different mitochondrial genes (*rns* or *coxI*). Each *Prasinoderma* intron is fragmented into two pieces, forming at the RNA level a secondary structure that resembles those of its *cis*-spliced counterparts. As observed for other *trans*-spliced group I introns, the breakpoint of the first intron maps to the variable loop L8, whereas that of the second is uniquely located downstream of P9.1. The breakpoint In each *Prasinoderma* intron corresponds to the same region where the open reading frame (ORF) occurs when present in *cis*-spliced orthologs. This correlation between the intron breakpoint and the ORF location in *cis*-spliced orthologs also holds for other *trans*-spliced introns; we discuss the possible implications of this interesting observation for *trans*-splicing of group I introns.

## Introduction

Group I and group II introns are mobile genetic elements frequently encountered in mitochondrial and plastid genomes [1]. They propagate to cognate and ectopic sites via homing and transposition processes. When inserted within genes, these selfish elements must be spliced following transcription such that the disrupted RNA function, e.g. mRNA, rRNA or tRNA, is properly restored. Group I and group II introns are generally capable of self-splicing through a series of transesterification reactions, which can be further facilitated *in vivo* by a maturase encoded within an open reading frame (ORF) present in the intron or by a splicing factor encoded elsewhere in the organelle genome or the nuclear genome [2], [3]. In some cases, external splicing factors are no longer accessory, being required usually in response to strong deviations from the respective canonical structures of these introns (e.g. [4], [5]).

Group I and group II introns can be found in *cis* or in *trans* configuration [6]. In *trans* configuration, the intron is split into non-adjacent pieces that are often far apart in the genome and located on different strands. These intron pieces flanked by exon sequences must interact at the RNA level to produce a functional intron structure that allows splicing to take place; the separate primary transcripts derived from the individual exon sequences are joined and ligated after assembly and splicing of the flanking intron sequences. While *trans*-spliced group II introns have been known for decades [7], [8], the first *trans*-spliced group I introns were reported in 2009 [9], [10], with only a few additional cases documented since their discoveries [11]–[14]. *Trans*-spliced group I introns are widely distributed across lineages and have been found within fungi [12], [13], placozoan animals [9], lycophytic plants [10], [11] and a chlorophyte green alga [14]. The nine currently known *trans*-spliced group I introns are restricted to the *cox1* and *rns* genes in mitochondria and examples of *trans*-spliced introns in the plastid have yet to be identified. All reported *trans*-spliced group I introns display bipartite RNA structures, with one instance hypothesized to use an helper RNA fragment to guide splicing [12]. In the *trans*-spliced introns whose secondary structures have been predicted, the junction between the 5′ and 3′ fragments, i.e. the breakpoint, is usually located in the loop subtending the base-paired region P8 (L8) [9], [13], [14]; however, the orthologous *cox1* introns found in the lycophytes *Isoetes engelmannii/Selaginella moellendorffii* are uniquely split in the L9 loop [10], [11]. Note here that the core structure of group I introns consists of a number of mandatory (P1, P3, P4, P5, P6, P7, P8 and P9) and optional (P2, P3.1, P6a, P7.1, P7.2, P9.2 and P9.3) base-paired regions (reviewed in [15], [16]). When present, intronic ORFs coding for homing endonucleases (LAGLIDADG, GIY-YIG, H-N-H, His-Cys box, or PD-(D/E)-XK; reviewed in [17]) are located within one of the variable loops subtending base-paired regions and sometimes extend across adjacent pairings. In rare cases (e.g. [14]), more than one ORF can be found in a single intron, each located in distinct loops and coding for distinct endonucleases.

In the course of investigating the mitochondrial genome of *Prasinoderma coloniale* CCMP 1220, a marine green alga belonging to the prasinophyte clade VI (Prasinococcales; [18], [19]) and for which little is known at the molecular level, we stumbled upon two interesting examples of *trans*-spliced group I introns. In the present study, we present the mitochondrial genome of *Prasinoderma coloniale*, compare it with four other prasinophyte mitochondrial DNAs (mtDNAs), and report the characteristics of its *trans*-spliced group I introns. The *Prasinoderma* mitochondrial introns are the first *trans*-spliced introns reported within the *rnl* gene. One of these introns also provides the first example of a *trans*-spliced group I intron split in the region downstream of P9.1. We show that the breakpoint in the bipartite RNA structure of each *Prasinoderma* intron corresponds to the same region that contains an ORF in *cis*-spliced relatives at the same cognate site. This correlation between the intron breakpoint and the ORF location in *cis*-spliced orthologs also holds for other *trans*-spliced introns.

## Results

*Prasinoderma coloniale* CCMP1220 belongs to one of the deepest prasinophyte lineages. The prasinophytes are paraphyletic, forming at least seven lineages (also known as clades I through VII) at the base of the Chlorophyta [18]. Some lineages display picoplanktonic species (i.e. organisms with a diameter of less than 3 µm), thus providing the opportunity to study the consequences of cell reduction on genome architecture. The small coccoid green alga *Prasinoderma* represents the Prasinococcales (clade VI), a lineage that had not been previously sampled for organelle genome studies. The complete mitochondrial genome sequence of *Prasinoderma* was compared with those of four other prasinophytes representing three distinct lineages: *Ostreococcus tauri* (clades II) [20], *Micromonas pusilla* (clade II) [21], *Nephroselmis olivacea* (clade III) [22] and *Pycnococcus provasolii* III (clade V) [23]. All four, except the flagellate *Nephroselmis*, are picoplanktonic prasinophytes, with *Ostreococcus* being the smallest free-living eukaryote described to date.

### Comparison of the *Prasinoderma* mtDNA with Other Prasinophyte Genomes

**Figure 1:**
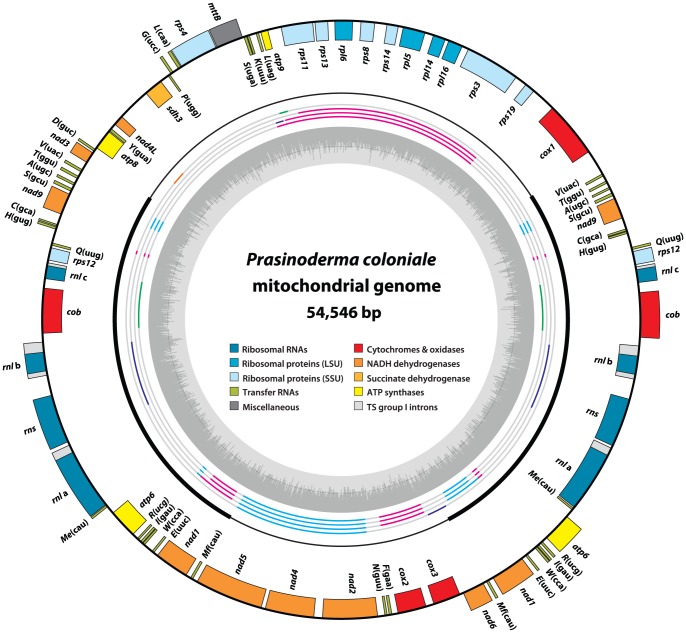
Physical map of the *Prasinoderma* mitochondrial genome. Genes and introns are represented by filled boxes. Genes on the outside are transcribed clockwise, whereas genes on the inside are transcribed counterclockwise. Colors are attributed according to the categories in the inner legend; TS group I introns refers to *trans*-spliced group I introns. *Outermost inner ring:* Inverted repeats and single copy regions are displayed by thick and thin black arcs, respectively. *Color-coded middle rings*: Syntenies between *Prasinoderma* and other prasinophyte mtDNAs. From the inside to the outside are shown the comparisons with *Ostreococcus*, *Micromonas*, *Nephroselmis* and *Pycnococcus*. Highly conserved clusters are shown in cyan and magenta, whereas clusters conserved only between *Prasinoderma* and one other prasinophyte are shown in green, purple or orange. Regions featuring no conserved clusters are shown in light gray. *Inner ring*: G+C percentages calculated with OGDRAW [45]; light gray, A+T; dark gray, G+C.

The *Prasinoderma* mitochondrial genome [GenBank:KF387569] maps as a 54,546 bp-long single circular molecule and features two copies of a large inverted repeat (14,364 bp each) encompassing 53% of the genome (Figure 1). While inverted repeats are usually rare in mitochondrial genomes, this feature is not unique to *Prasinoderma* mtDNA and has been previously identified in the two prasinophytes representing clade II (Table 1). With an A+T content of 54.2%, the *Prasinoderma* mtDNA presents a surprisingly low bias towards these nucleotides, differing from the genomes of other prasinophytes (Table 1) and also from those of most other green algae analyzed so far by a noticeable margin [24]. Only a few exceptional GC-biased green algal and land plant mtDNAs have been documented [25], [26]. As typically observed for AT-biased organelle genomes, the percentage of adenosines and thymidines in the intergenic regions of the *Prasinoderma* genome (59.6%) is slightly higher than that found in genes (52.3%).

**Table 1:** Main features of the prasinophyte mitochondrial genomes compared in this study.

	*Ostreococcus*	*Micromonas*	*Nephroselmis*	*Pycnococcus*	*Prasinoderma*
Genome feature	II^a^	II	III	V	VI
Size (bp)	44,237	47,425	45,223	24,321	54,546
% A+T	61.8	65.4	67.2	62.2	45.8
Inverted repeat	+	+	−	−	+
Size (bp)	9,772	12,234			14,364
% Genome	44.2	51.6			54.2
Genes					
Number^b^	63 (78)	63 (79)	66 (70)	36 (36)	55 (74)
% Genome c	89.9	84.8	80.2	87.7	73.8
Introns^d^					
Group I	0 (0)	0 (0)	4 (0)	0 (0)	0 (2)
Group II	0 (0)	0 (0)	0 (0)	0 (0)	0 (0)
Repeats^e^					
Number	34	0	42	242	2141
% Genome	0.1	0	0.1	1.0	3.9
Accession Number	NC_008290	NC_012643	NC_008239	NC_013935	KF387569

a The Roman number refers to the phylogenetic clade identified by Guillou et al [18].

b The total number of unique genes is indicated outside the parentheses. The number inside the parentheses includes duplicated genes and intronic ORFs.

c Conserved genes (unique and duplicated), introns and intronic ORFs were taken into account.

d The total numbers of *cis*-spliced and *trans*-spliced introns are indicated outside and inside the parentheses, respectively.

e Nonoverlapping repeat elements were mapped on each genome with RepeatMasker using the repeats ≥30 bp identified with REPuter.

The *Prasinoderma* mitochondrial genome encodes a set of 55 unique genes, 20 of which (including both rRNA genes) are present in the large inverted repeat. This gene repertoire is intermediate between those of the gene-rich genomes of *Ostreococcus tauri*, *Micromonas pusilla* and *Nephroselmis olivacea* (clades II and III) and that of the gene-poor genome of *Pycnococcus provasolii* (clade V) (Table 1). Unlike its four prasinophyte homologs, *Prasinoderma* mtDNA features *sdh3*, the gene coding for the third subunit of succinate dehydrogenase. The subset of 16 genes present in the clade II/III genomes but absent from *Prasinoderma* mtDNA are also missing in the gene-poor genome of *Pycnococcus* (Table 2). These differences do not reflect gradual gene losses across prasinophyte lineages, as mapping of the presence/absence of mitochondrial genes on the phylogenetic tree reported by Guillou et al. [18] rather suggests that independent gene losses occurred in clades V and VI (data not shown). Note that, in contrast to *Pycnococcus* mtDNA, all protein-coding genes in the *Prasinoderma* genome use the standard genetic code.

**Table 2:** Gene repertoires of prasinophyte mtDNAs.

	*Ostreococcus*	*Micromonas*	*Nephroselmis*	*Pycnococcus*	*Prasinoderma*
Gene^a^	II^b^	II	III	V	VI
*atp1*	+	−	+	−	−
*atp4*	+	+	+	−	−
*mttB*	+	+	+	−	+
*nad7*	+	+	+	−	−
*nad9*	+	+	+	−	+
*nad10*	+	+	+	−	−
*rnpB*	−	−	+	−	−
*rpl5*	+	+	+	−	+
*rpl6*	+	+	+	−	+
*rpl10*	−	−	+	−	−
*rpl14*	+	+	+	−	+
*rpl16*	+	+	+	−	+
*rps2*	+	+	+	−	−
*rps7*	+	+	+	−	−
*rps8*	+	+	+	−	+
*rps10*	+	+	+	−	−
*rps11*	+	+	+	−	+
*rps13*	+	+	+	−	+
*rps14*	+	+	+	−	+
*rps19*	+	+	+	−	+
*rrn5*	−	−	+	−	−
*sdh3*	−	−	−	−	+
*trnA(ugc)*	+	+	+	−	+
*trnF(gaa)*	+	+	+	−	+
*trnG(gcc)*	+	+	−	−	−
*trnG(ucc)*	+	+	+	−	+
*trnI(cau)*	−	+	+	−	−
*trnK(uuu)*	+	+	+	−	+
*trnL(caa)*	−	−	−	−	+
*trnL(gag)*	+	+	−	−	−
*trnL(uaa)*	+	+	+	−	−
*trnR(acg)*	+	+	+	−	−
*trnR(ucg)*	−	−	+	−	+
*trnR(ucu)*	+	+	+	−	−
*trnT(ggu)*	+	+	+	−	+

a Only the genes that are missing in one or more genomes are indicated. A total of 35 genes are shared by all compared mtDNAs: atp6, 8, 9, cob, cox1, 2, 3, nad1, 2, 3, 4, 4L, 5, 6, rnl, rns, rps3, 4, 12, trnC(gca), D(guc), E(uuc), H(gug), I(gau), L(uag), Me(cau), Mf(cau), N(guu), P(ugg), Q(uug), S(gcu), S(uga), V(uac), W(cca), Y(gua).

b The Roman number refers to the phylogenetic clade identified by Guillou et al [18].

Conserved genes represent 73.8% of the *Prasinoderma* genome; this is the least densely packed mtDNA among the prasinophyte mitochondrial genomes sequenced so far (Table 1). This lower coding density is correlated with an increased level of small repeated sequences, which accounts for almost 4% of the total genome size (Table 1). Repeated sequences are not uncommon in green algal mitochondrial genomes, being prevalent in ulvophycean lineages [27], [28].

In terms of synteny, the *Prasinoderma* mitochondrial genome shares a number of gene clusters with other prasinophyte mtDNAs (Figure 1; inner rings). The 5′-*nad5*-*nad4*-*nad2*-3′ gene cluster is conserved between all these genomes, whereas the 5′-*cox2*-*cox3*-3′, 5′-*nad1*-*trnMf*(cau)-3′, 5′-*trnR*(ucg)-*trnI*(gau)-3′, 5′-*trnS*(gcu)-*trnA*(ugc)-*trnT*(ggu)-3′ and 5′-*rps11*-*rps13*-*rpl6*-*rps8*-*rps14*-*rpl5*-*rpl14*-*rpl16*-*rps3*-*rps19*-3′ clusters have been eroded only in the *Pycnococcus* lineage. The genes comprised in the latter ribosomal protein cluster are missing entirely from the *Pycnococcus* mtDNA [23].

**Figure 2:**
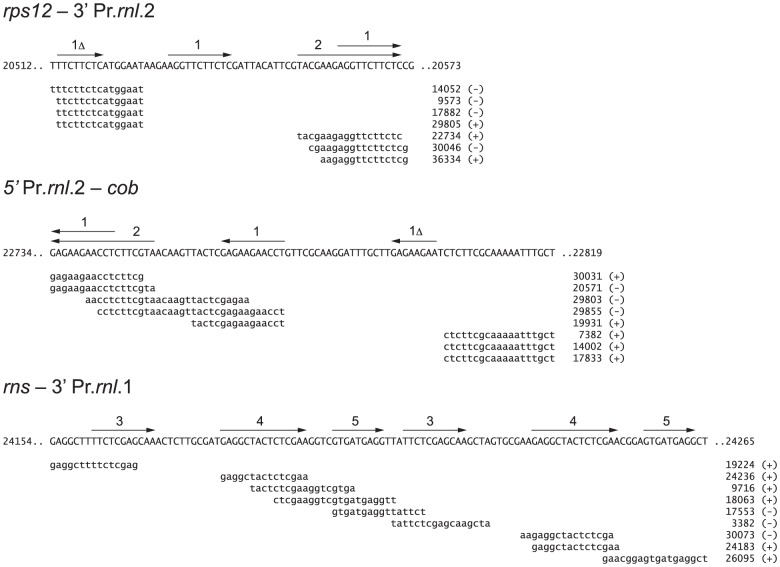
Small repeats identified in three of the four *Prasinoderma* mitochondrial intergenic regions bordered by *rnl* intron fragments. The figure shows the repeated sequences ≥15 bp located in each of these intergenic regions (top sequence) as well as elsewhere in the *Prasinoderma* mtDNA. The latter repeats are aligned against the intergenic sequence found in copy A of the large inverted repeat (positions 17,080 to 31,443), with the numbers indicating the coordinates of the genome sequence corresponding to the 5′ ends of the repeats. A plus or minus sign is used to indicate the DNA strand containing each repeat, with the plus sign denoting the strand whose sequence is reported in GenBank accession KF387569. To simplify the figure, the repeats located in copy B of the inverted repeat (positions 40,182 to 54,545) were not shown. Numbered arrows indicate the repeats (8 to 18 bp) present in multiple copies within each intergenic region as well as the repeats shared between the intergenic regions bordered by Pr.*rnl*.2 fragments. Repeats ≥15 bp were detected using the REPuter 2.74 program [44] with the options -f -p -l 15 -allmax; no repeats were found in the intergenic region delimited by 5′ Pr.*rnl*.1 and *rns*.

Prasinophyte mitochondrial genomes are generally poor in introns (Table 1). Of the five prasinophyte mtDNAs sequenced so far, only those of *Prasinoderma* and *Nephroselmis* harbor these elements, with two and four group I introns found in these mtDNAs, respectively. Both *Prasinoderma* group I introns and three of the *Nephroselmis* introns reside in the large subunit rRNA gene (*rnl*), whereas the remaining *Nephroselmis* intron is located in *cob*. Unlike their *Nephroselmis* counterparts, the two *Prasinoderma* mitochondrial introns, named hereafter Pr.*rnl*.1 and Pr.*rnl*.2, are discontinuous. Each of these introns is fragmented into two non-adjacent pieces, thus accounting for the three distinct coding regions observed for the *rnl* gene. The *rnl* a and *rnl* b exons are separated from one another by *rns*, a gene encoded on the same DNA strand as the latter exons; in contrast, the third piece of *rnl* lies on the opposite strand between *cob* and *rps12* (Figure 1). In three of the four intergenic regions bordered by intron fragments, small repeated sequences ≥15 bp in size were identified near the intron breakpoint (Figure 2); they are present at other locations in the large inverted repeat and/or elsewhere in the mitochondrial genome. In addition, within each of these intergenic regions, we detected repeats ≤15 bp that are present in more than one copy and in direct orientation (repeats 1 and 3-5 in Figure 2). Note that repeats 1 and 2 are found in both intergenic regions bordered by Pr.*rnl*.2 sequences.

### Features of the Two *Prasinoderma* Mitochondrial *Trans*-spliced Group I Introns

**Figure 3:**
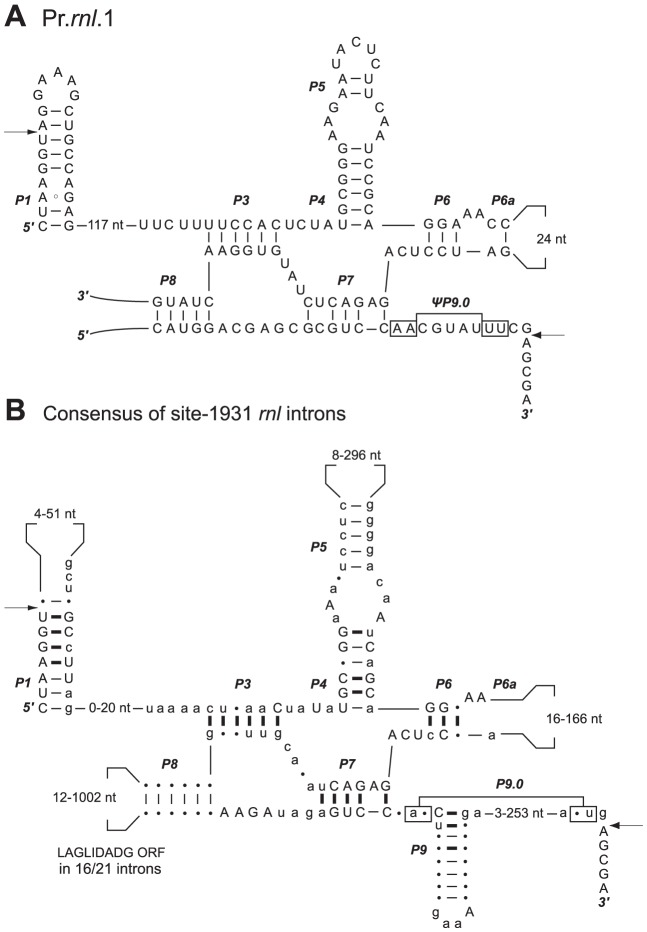
*Prasinoderma* Pr.*rnl*.1 intron and its relatives. Comparison of the predicted secondary structure of Pr.*rnl*.1 (*A*) with the consensus structure derived from organellar *cis*-spliced introns at the same cognate site (*B*). This consensus was generated using the structures of the 21 site-1931 mitochondrial and plastid introns in the Group I Intron Sequence and Structure Database [33]. Introns are displayed according to Burke et al [46]. Highly conserved residues (in all 21 introns) and less conserved residues (in 15 to 20 introns) are shown in uppercase and lowercase characters, respectively; the other residues are represented by dots. Conserved base-pairings in all introns and in 15 to 20 introns are denoted by thick and thin dashes, respectively. The P9.0 base-pairing is represented according to Cech [15]. Numbers inside the loops indicate the size variations of these loops. Splice sites between intron and exon junctions are indicated by arrows.

**Figure 4:**
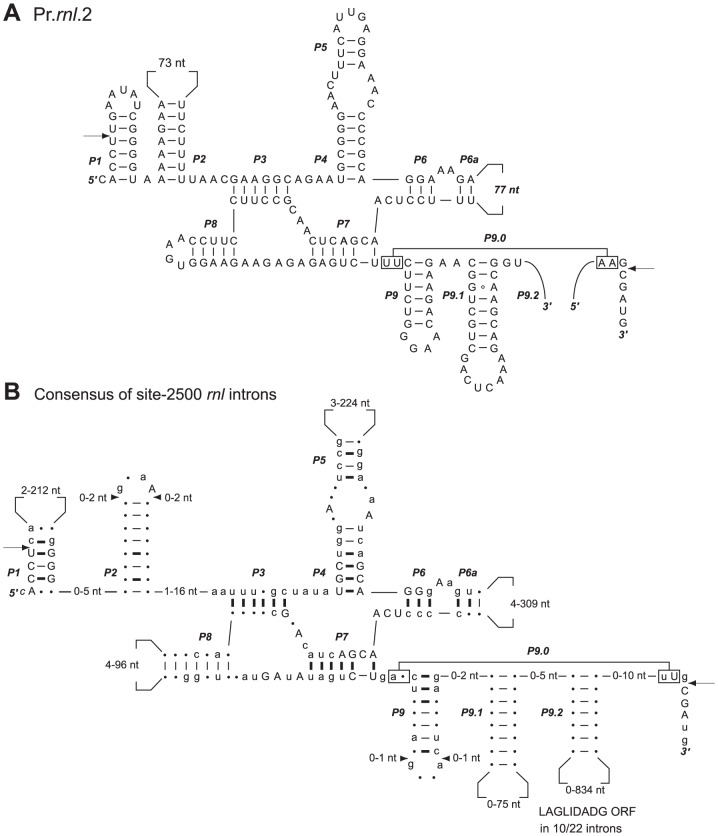
*Prasinoderma* Pr.*rnl*.2 intron and its relatives. Comparison of the predicted secondary structure of Pr.*rnl*.2 (*A*) with the consensus structure derived from organellar *cis*-spliced introns at the same cognate site (*B*). This consensus was generated using the structures of the 22 site-2500 mitochondrial and plastid introns in the Group I Intron Sequence and Structure Database [33]. Introns are displayed according to Burke et al [46]. Highly conserved residues (in all 22 introns) and slightly less conserved residues (in 16 to 21 introns) are shown in uppercase and lowercase characters, respectively; the other residues are represented by dots. Conserved base-pairings in all introns and in 16 to 21 introns are denoted by thick and thin dashes, respectively; the others are represented by dots. The P9.0 base-pairing is represented according to Cech [15]. Arrowheads point to sites of insertions/deletions. Numbers inside the loops indicate the size variations of these loops. Splice sites between intron and exon junctions are indicated by arrows.

**Figure 5:**
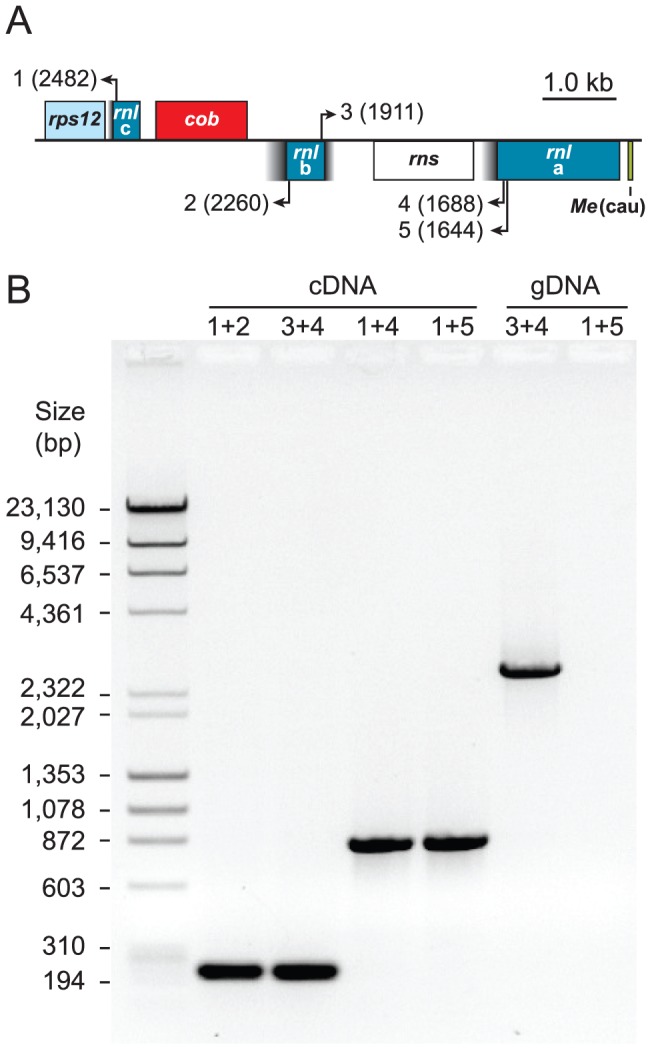
Confirmation of group I intron *trans*-splicing by RT-PCR analysis. (*A*) Genomic configuration of the *rnl* exons in *Prasinoderma* mtDNA. *Trans*-spliced group I intron sequences are shown as black-to-gray gradient boxes. Primer locations are indicated by numbered arrows (see methods for primer sequences); the numbers in parentheses denote the nucleotide positions corresponding to the 5’ ends of the primers on the predicted *rnl* gene product, *i.e.* the RNA species derived from the three *rnl* exon sequences. Coding regions shown above or below the horizontal line are transcribed to the right or to the left, respectively. (*B*) Electrophoretic analysis of PCR products. PCR assays were carried out on cDNA or genomic DNA (gDNA), with the numbers above the gel lanes indicating the combinations of primers used. The sizes of the amplicons derived from the PCR assays on cDNA are entirely consistent with the hypothesis that two events of *trans*-splicing must occur to produce the large subunit RNA sequence. The results obtained for the two PCR assays on gDNA are also those expected: the assay using primers 3 and 4 yielded an amplicon with the size predicted by the genome map, whereas the assay using primers 1 and 5 produced no amplicon because both primers point toward the same direction. The identities of all amplicons were confirmed by DNA sequencing.

The two pieces of each *Prasinoderma trans*-spliced group I intron must be assembled in *trans* at the RNA level to produce the group I intron structure (Figures 3A and 4A). We have confirmed by RT-PCR experiments that both *Prasinoderma* introns are spliced properly and that the *rnl* gene sequence is contiguous at the RNA level (Figure 5) despite being encoded by three distinct pieces located on opposite strands at the DNA level (Figure 1).

**Figure 6:**
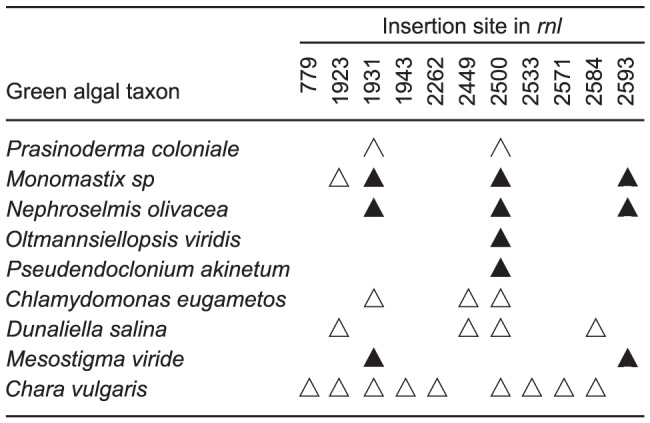
Intron insertion sites in the mitochondrial *rnl* genes of selected green algae. *Cis*- and *trans*-spliced introns are shown by triangles and broken triangles, respectively. When present, intronic ORFs are shown by filled triangles. Intron insertion sites are given relative to the *E. coli* 23S rRNA; for each site, the position corresponding to the nucleotide immediately preceding the intron is reported. Accession numbers for the *rnl* sequences represented are as follows: *Prasinoderma coloniale*, KF387569; *Monomastix* sp, KF060939; *Nephroselmis olivacea*, NC_008239; *Oltmannsiellopsis viridis*, NC_008256; *Pseudendoclonium akinetum*, NC_005926; *Chlamydomonas eugametos*, NC_001872; *Dunaliella salina*, NC_012930; *Mesostigma viride*, NC_008240; and *Chara vulgaris*, NC_005255.

The insertion sites of Pr.*rnl*.1 and Pr.*rnl*.2 are not unique to *Prasinoderma*. The first and second introns in the *Nephroselmis* mitochondrial *rnl* are inserted at exactly the same sites (Figure 5), which correspond to positions 1931-1932 and 2500-2501 in the 23S rRNA sequence of *Escherichia coli* [29]. Furthermore, these insertion sites are also occupied by group I introns in the mitochondrial and plastid large subunit RNA genes of other green algae (e.g. see Figure 5) and a variety of other organisms, including bacteria (e.g. [27], [28], [30]-[33]). While Pr.*rnl*.1 and Pr.*rnl*.2 contain no ORF, numerous *cis*-spliced introns at the same cognate sites encode a LAGLIDADG homing endonuclease (e.g. see Figure 6).

The predicted RNA secondary structures of Pr.*rnl*.1 and Pr.*rnl*.2 are consistent with the consensus structures derived from the mitochondrial and plastid *cis*-spliced introns inserted at cognate sites (Figures 3 and 4), all of which are IB4 introns [33]. However, in contrast to its *cis*-spliced relatives, the Pr.*rnl*.1 intron does not display sufficient nucleotides at its 3′end to form the canonical P9 pairing. The site at which this intron is split corresponds to the L8 loop. In Pr.*rnl*.2, the breakpoint is located in the segment comprised between P9.1 and the 3′-terminus, a feature unique among the *trans*-spliced group I introns examined to date. Interestingly, each *Prasinoderma trans*-spliced intron is split in the same loop that contains the ORF in *cis*-spliced orthologs. We also found that there is a correspondence between the breakpoint and the ORF location in the case of the previously described *trans*-spliced group I introns that have known *cis*-spliced relatives at cognate sites (Table 3).

**Table 3:** Previously reported *trans*-spliced group I introns in *cox1* that have *cis*-spliced relatives containing an ORF at cognate sites.

*Trans*-spliced *cox1* introns			*Cis*-spliced *cox1* orthologs with ORF			
Taxon^a^	Accession	Breakpoint	Taxon	Accession	ORF location	ORF type
**Site 762** b						
*Helicosporidium* sp.	NC_017841	L8	*Prototheca wickerhamii* 3	NC_001613	L8	LAGLIDADG
			*Chlorokybus atmophyticus* 1	NC_009630	L8	LAGLIDADG
			*Nitella hyalina* 3	NC_017598	L8	LAGLIDADG
**Site 773**						
*Tricoplax adhaerens* 4	NC_008151	L8	*Klebsormidium* spec. 5	KF060942	L8	LAGLIDADG
*Gigaspora margarita* 4	NC_016684	L8	*Emericella nidulans* 3	X00790	L8	LAGLIDADG
*Gigaspora rosea* 4	NC_016985	L8	*Schizosaccharomyces pombe* 2	NC_001326	L8	LAGLIDADG
**Site 1338**						
*Isoetes engelmannii* 6	FJ390841	L9	*Chlorogonium elongatum* 2	Y13644	L9	GIY-YIG
*Selaginella moellendorffii* 7	JF338145	L9				

a When multiple introns are present, a number following the taxon name designates the intron number.

b Each of the three intron insertion sites is given relative to the *cox1* gene of *Mesostigma viride*; for each site, the position immediately preceding the intron is given.

## Discussion

Sequencing of the *Prasinoderma* mtDNA was undertaken as part of a larger project aimed at studying the diversity of the mitochondrial genome in prasinophytes, inferring the ancestral state of this genome in the Chlorophyta, and examining the potential consequences of cell reduction on genome architecture. Previous sampling of four prasinophytes representing three of the seven major clades recognized for these green algae (clades I, II and V) had revealed important variations at the level of mitochondrial genome size, gene content, gene density, and overall genome structure among lineages [20], [21], [23]. The newly sequenced mitochondrial genome of the picoplanktonic alga *Prasinoderma*, a representative of clade VI, also differs substantially from its counterparts (Table 1), including the three other picoplanktonic prasinophytes previously examined (*Ostreococcus*, *Micromonas* and *Pycnococcus*). The *Prasinoderma* genome has retained more genes than its *Pycnococcus* homolog but has lost many compared to the *Ostreococcus, Micromonas and Nephroselmis* mtDNAs, yet its genome size is the largest known among prasinophytes. Moreover, contrary to the genomes of the other three picoplanktonic prasinophytes, which are very tightly packed with genes and lack introns, that of *Prasinoderma* is much less compact than the *Nephroselmis* genome and like the latter contains introns. The comparative data reported here thus highlight differences in the types and extent of mtDNA changes that accompanied cell reduction in clades I, V and VI although these three lineages all display a reduced gene content.

The finding of two introns in the *Prasinoderma* mtDNA was not surprising given the low gene density of this genome; however, the discovery that both are *trans*-spliced group I introns in the *rnl* gene was very unexpected. To our knowledge, these introns are the first *trans*-spliced group I introns reported in the *rnl* gene. In green algae/land plants, *trans*-splicing of group I introns had previously been reported only for the mtDNAs of the parasitic trebouxiophyte *Helicosporidium* [14] and the lycophytic plants *Isoetes* and *Selaginella* [10], [11], And, as is the case for most other known *trans*-spliced group I introns, *cox1* was the gene interrupted.

While *trans*-spliced group I introns are still fairly new molecular oddities, we expect that these catalytic ribozymes will be encountered more often in the future. This is due to the dramatically improved DNA sequencing capabilities that allow sampling both in depth and coverage of previously uninvestigated lineages at an unprecedented pace. It is perhaps not surprising that all of the reported examples of *trans*-spliced group I introns are located in the well-known *cox1* and *rns* and, in this study, in the *rnl* gene. The main reason is that these genes, in particular *cox1* and *rnl*, are often rich in introns and contain numerous potential intron insertion sites. Moreover, because the products of these genes are well conserved and essential for mitochondrion function, their partial or total absence from annotations is looked upon with suspicion. In contrast, divergent genes are often hard to analyze and thus annotation errors are more likely to be left unnoticed. Finding introns in such genes either in *cis* or *trans* configuration can be far from trivial, and when these introns are further split into distinct pieces jumbled across a whole genome, the complexity of this task is compounded.

Intuitively, the conversion of an intron from a *cis* to a *trans* configuration is rather straightforward and implies one or more recombination events in a segment of the intron that is malleable enough to accommodate the disruption. Therefore, variable loops containing expanded stretches of DNA between conserved pairings are the most obvious targets for recombination. Accordingly, all of the *trans*-spliced group I introns reported so far that have *cis*-spliced orthologs are broken at the same variable region as the one featuring the ORF in their *cis*-spliced relatives (Figures 3 and 4 and Table 3). This correlation between the breakpoint of the *trans*-spliced intron and the ORF location in *cis*-spliced relatives also applies to group II introns [34], [35].

While the apparent preference for ORF-containing loops over other variable loops as the site of *trans*-splicing in group I introns may result from a low sampling artefact, it could also reflect the mechanism underlying the *cis* to *trans* conversion of these elements. Indeed, ORFs coding for homing endonucleases are often similar in sequence and can in principle serve as hotspots for semi-homologous recombination events, thereby increasing the probability of fracturing these intron regions. However, we found no intronic ORFs nor free-standing ORFs coding for homing endonucleases in the *Prasinoderma* mitochondrial genome. Instead, further investigation of the mtDNA regions near the intron breakpoints disclosed short dispersed repeats as potential recombination targets (Figure 2). Interestingly, a number of *trans*-spliced group II introns, in particular flowering plant mitochondrial introns, appear to have been generated by homologous recombination across short repeats [36]–[38], although it is also possible that recombination occurred between intronic ORFs and related ORFS located elsewhere in the mitochondrial genome [38].

Aside from the DNA rearrangements discussed above, at least two major conditions must be met for successful events of *cis* to *trans* intron conversion. First, in the case of a bipartite *trans*-spliced intron, the two intron pieces together with their attached exons must be transcribed independently and second, the intron segments must be spliced properly, as failure to do so would result in a truncated product likely to be deleterious, if not lethal, to the fitness of the cell. Therefore, following recombination, the newly formed 3′ segment of the intron must either acquire its own promoter or be positioned in such a way as to be co-transcribed with the upstream gene. However, even if the two intron sections and their flanking exons are transcribed properly, there is no guarantee that interaction of the resulting precursor RNAs via base-pairings of the intron fragments will result in an intron structure that will enable the self-splicing reaction to occur; one or more external accessory factor(s) acting as a *de facto* maturase might be required to yield the productive structure necessary for splicing. Reliance on many nuclear-encoded splicing factors (at least 14) has been demonstrated for the tripartite *trans*-spliced group II intron found in the chloroplast of the green alga *Chlamydomonas reinhardtii* [2], [4], [7]. In the case of the *Prasinoderma* Pa.*rnl*.1 intron, it is intriguing that the two fragments linked to the flanking exons cannot form the typical secondary structure expected for a group IB intron (Figure 2). Indeed, the potential secondary structure we modelled from these pieces is very unusual in lacking P9, an essential base-paired region. We cannot eliminate the possibility that a third intron piece yet to be discovered in the *Prasinoderma* mitochondrial genome supplies the missing P9 region; in the absence of such a piece, splicing of Pr.*rnl*.1 would likely depend on external accessory factor(s).

## Conclusions

The comparative genome analysis presented here underscores the high variability in mtDNA architecture among prasinophyte lineages. The newly sequenced mitochondrial genome of the picoplanktonic green alga *Prasinoderma* has several unique characteristics, including the presence of two *trans*-spliced group I introns in the *rnl* gene. Sampling of other prasinophyte lineages should provide further insights into the range of mtDNA variations seen in these basal chloroplast lineages and could also help deepen our understanding of how *trans*-spliced introns arise.

## Materials and Methods

### Strain, Culture and DNA Extraction

*Prasinoderma coloniale* strain CCMP 1220 was obtained from the Provasoli-Guillard National Center for Marine Algae and Microbiota (Maine, USA). *Prasinoderma* cells were cultured in K medium [39] at 18°C under 12h-light/-12h-dark cycles and subpassaged every two weeks. Total cellular DNA was extracted as described in Turmel et al [22]. A+T-rich organellar DNA was separated from nuclear DNA by CsCl-bisbenzimide (1.67 g/ml CsCl, 200 µg/ml bisbenzimide) isopycnic centrifugation as described previously [22], and the resulting gradient was fractionated into 40 fractions (120 µs each) using a Density Gradient Fractionation System (Brandel, Gaithersburg, MD). DNA from each of the 20 lowest density fractions was recovered by precipitation with ethanol and dissolved in TE buffer. Aliquots of these DNA samples were digested with EcoRI and their restriction patterns visualized on an agarose gel. Fractions displaying digestion patterns of low complexity DNA were selected for sequencing.

### Genome Sequencing, Assembly and Annotation

A shotgun library of *Prasinoderma* A+T-rich organellar DNA (700 bp fragments) was constructed using the GS-FLX Titanium Rapid Library Preparation Kit from Roche 454 Life Sciences (Branford, CT, USA). Construction of this library as well as 454 GS-FLX DNA Titanium pyrosequencing (one eight of a run) were carried out by the Plate-forme d′Analyses Génomiques (Université Laval, Québec, Canada). The resulting reads were assembled with gsAssembler 2.5 from the Roche GS Data Analysis Software package (Branford, CT, USA). Contigs were visualized, linked, edited and polished using the CONSED 22 package [40]. Ambiguous regions in the assemblies were amplified by PCR with primers specific to the flanking sequences. Purified PCR products were sequenced using Sanger chemistry with the PRISM BigDye terminator cycle sequencing ready reaction kit (Applied Biosystems, Foster City, CA, USA) by the Plate-forme d′Analyses Génomiques on an ABI model 373 DNA sequencer (Applied Biosystems). Genes and ORFs were identified on the final assembly (107× minimum coverage) using a custom-built suite of bioinformatics tools as described previously [41]. tRNA genes were localized using tRNAscan-SE [42]. Intron boundaries were determined by modeling intron secondary structures according to Michel and Westhof [43] and by comparing intron-containing genes with intronless homologs. To estimate the proportion of repeated sequences in the *Prasinoderma* mtDNA, repeats ≥30 bp were retrieved using REPFIND of the REPuter 2.74 program [44] with the options -f (forward) -p (palindromic) -l (minimum length = 30 bp) -allmax and then were masked on the genome sequence using REPEATMASKER (http://www.repeatmasker.org/) running under the Crossmatch search engine (http://www.phrap.org/).

### RNA extraction and RT-PCR reactions

Total RNA from *Prasinoderma* was extracted from cells ground in liquid nitrogen with the Qiagen RNeasy Midi kit (Mississauga, Ontario, Canada) as described in Turmel et al [23]. To confirm that mitochondrial *rnl* transcripts undergo *trans*-splicing and also to confirm the insertion positions of the *trans*-spliced introns, RT-PCR reactions were performed on the DNA-free RNA using the Qiagen One-Step RT-PCR kit with the following primers: 1) 5′-ACCAAACTGTCTTACGACGTTC-3′, 2) 5′-ATACTGAACCGGAGTTTCCTTG-3′, 3) 5′-CTTCAATTTCACCGAGTCCATG-3′, 4) 5′-ACAGGTCTCTGCAAAGTCGAAG-3′, 5) 5′-GTGAAGTCGCAGAAAATTGTGG-3′ (for their genomic locations, consult Fig. 4A). The RT-PCR products were sequenced using Sanger chemistry as described above.
